# On-Call Simulation: A One-Day Comprehensive Simulation of Clinical Practice for Final-Year Medical Students

**DOI:** 10.7759/cureus.74555

**Published:** 2024-11-27

**Authors:** Omar Desouky, Natasha Lawes, Tom Hunter, Nowera Zafar, Wendy Whitehead

**Affiliations:** 1 Department of Undergraduate Education, Royal Blackburn Hospital, Blackburn, GBR

**Keywords:** emergency simulation, medical edu, medical student teaching, simulation based training, simulation course, simulation design, simulation in medical education, skills and simulation training, student education, teaching by simulation

## Abstract

Introduction

Transitioning from a medical student to a foundation doctor presents numerous challenges, particularly in managing on-call duties that require quick decision-making, clinical skills, and prioritisation under pressure. The Simulation On-Call (SOC) program was developed as a one-day, immersive simulation event to equip final-year medical students with the skills and confidence needed for these responsibilities.

Methods

The SOC program is an annual event held for final-year medical students at the Royal Blackburn Hospital, Blackburn, UK. It is conducted over five days, with 80 final-year medical students rotating through nine stations that replicated common on-call scenarios. Each station required students to address critical medical situations, such as managing an acutely unwell patient, electrolyte abnormalities, and referring and communicating with colleagues, while managing realistic interruptions through a "bleep" system. Pre- and post-event questionnaires, featuring 25 Likert-scale items, were disseminated to assess student feedback and self-reported confidence across competencies, including communication, clinical decision-making, and task prioritisation. A retrospective review and analysis of the feedback was performed to assess the impact of the SOC program by assessing the pre- and post-session questionnaires.

Results

Of the 80 participants, 70 completed the pre-event questionnaire, and 72 completed the post-event questionnaire. Statistical analysis revealed a significant increase in students’ self-reported confidence across all 25 Likert-scale items, with marked improvements in task prioritisation (rₛ = 0.74, p < 0.001), managing acutely unwell patients (rₛ = 0.60, p < 0.001), and escalation skills (rₛ = 0.49, p < 0.001). Effect sizes ranged from moderate to large, underscoring the practical impact of the Simulation On-Call program in enhancing preparedness and confidence for on-call responsibilities.

Conclusion

The SOC program provided a valuable, realistic, and supportive environment for final-year medical students to practice on-call responsibilities. The significant improvement in student confidence highlights the effectiveness of this program as a preparatory tool for foundation doctors. Future iterations will incorporate feedback to continually refine the simulation experience, ensuring its alignment with clinical demands.

## Introduction

The use of simulation in medical education has grown significantly as an effective approach to develop clinical competencies while ensuring patient safety [1,2]. Simulation-based training allows learners to practice technical and non-technical skills in controlled settings, reducing patient exposure to inexperienced practitioners during the early stages of their training [1,3]. This pedagogical approach has demonstrated its utility across various competencies, including procedure-based skills, communication, teamwork, and leadership [3-5]. Notably, simulation helps bridge the gap between theoretical knowledge and practical application, allowing medical students to experience realistic clinical scenarios that build confidence and preparedness for real-life situations [3,4,6]. Advancements in simulation technology, such as high-fidelity mannequins, virtual reality, and serious games, have further enhanced the realism and effectiveness of simulation-based education [7]. High-fidelity simulations provide immersive experiences that closely mimic clinical environments, enabling learners to develop critical thinking and decision-making skills [7]. Cook et al. conducted a meta-analysis demonstrating that technology-enhanced simulation training is associated with significant improvements in knowledge, skills, and behaviours compared to traditional educational methods [8]. Simulation-based mastery learning can directly improve patient care outcomes. A study by Barsuk et al. showed that simulation-based learning improved patient outcomes, such as reducing catheter-related bloodstream infections, highlighting the direct impact of simulation training on patient safety [9].

The transition from medical student to practising doctor is often abrupt and challenging. New graduates face a steep learning curve as they adapt to high-stakes clinical environments and assume responsibility for patient care [10,11]. Studies have shown that a significant proportion of new doctors feel underprepared for their roles, particularly in critical areas such as managing acutely unwell patients, prioritising tasks, and making rapid decisions under pressure [10-12]. The psychological toll of this transition is considerable, with high levels of stress, anxiety, and burnout reported among first-year doctors [13,14]. A systematic review by West et al. highlighted the effectiveness of interventions in reducing physician burnout, which is associated with improved quality of care and patient safety [15]. Efforts to address this challenge have led to the incorporation of preparatory programs and simulation-based training, which aim to improve readiness and reduce transitional stress [10,13,16].

One major area of difficulty for new doctors is the prioritisation of clinical tasks during on-call duties. A recent study emphasised the importance of prioritisation skills in clinical practice, noting that time management and task prioritisation are often inadequate in traditional medical curricula [17]. Murphy et al. highlighted the use of Stephen Covey’s Time Management Matrix Technique in workshops designed for medical students, which showed improved confidence in managing clinical tasks [18]. The inability to prioritise effectively can lead to delayed patient care, increased workload, and heightened stress, impacting both patient safety and the well-being of healthcare providers [19,20]. Addressing this skills gap is crucial, as prioritisation underpins effective clinical decision-making, especially in urgent care settings [17,19].

Simulation has been identified as a beneficial tool in enhancing prioritisation and decision-making skills in healthcare settings [21]. By providing a safe environment for learners to practice and make mistakes without compromising patient safety, simulation-based programs can focus on developing competencies critical to effective patient care [22,23]. Ingrassia et al. conducted a study using a desirability function to identify and prioritise procedural skills within a simulation curriculum, affirming the role of simulation in bridging competency gaps among medical students [22]. Moreover, simulation-based interventions focusing on teamwork and communication have proven valuable, aligning with the collaborative nature of healthcare environments and preparing students to function effectively within interdisciplinary teams [4,24]. Team training through simulation has been shown to improve team behaviours and patient outcomes, reinforcing its importance in medical education [24].

Another benefit of simulation-based training is cost-effectiveness. It has been identified as a cost-effective method when considering the potential reduction in medical errors and associated costs [9]. While initial investment in simulation technology and resources can be substantial, the long-term benefits, including improved clinical competence and patient safety, may offset these costs. Cook et al. found that technology-enhanced simulation training is associated with significant improvements in knowledge and skills compared to traditional methods, suggesting a favourable return on investment [8].

Despite these benefits, simulation cannot entirely replace the experience gained from real clinical encounters. It is most effective when integrated with practical experiences, such as assistantships or internships, allowing learners to translate simulated skills into real-world contexts [11,25]. Kneebone et al. proposed an innovative model combining simulation with clinical practice, emphasising the importance of contextual learning [25]. Integrating simulation into medical curricula as a transitional support strategy can significantly enhance new doctors’ preparedness, reducing the risks associated with the shift from supervised student to autonomous practitioner [19,26]. Understanding transitions as critically intensive learning periods underscores the need for comprehensive educational strategies that support learners during this critical phase [26].

Our aim was to evaluate the impact of a one-day, simulation-based "on-call" training program designed for final-year medical students. This program focused on immersing students in realistic on-call scenarios to assess its effects on their confidence, prioritisation skills, and overall preparedness for autonomous practice. Through structured assessments and feedback, we sought to understand how such targeted simulations could support final-year students in managing the demands of on-call responsibilities effectively.

## Materials and methods

The Simulation On-Call (SOC) program is an annual event held for final-year medical students at the Royal Blackburn Hospital, Blackburn, UK. The program simulated on-call scenarios commonly encountered in hospital settings, with the intent to immerse students in realistic, high-stakes clinical situations that mimic the clinical responsibilities they will face as newly qualified doctors. Pre- and post-event questionnaires, featuring 25 Likert-scale items, are disseminated to assess student feedback and self-reported confidence across various domains. For the 2023 iteration, a retrospective review and analysis of the feedback was performed to assess the impact of the SOC program by assessing the pre- and post-session questionnaires.

Simulation program design

The SOC program was designed to reflect real on-call duties. It included nine interactive stations, each replicating scenarios requiring prioritisation and decision-making under time pressure. Examples of scenarios included managing acutely ill patients, verifying death, challenging discussions with family members, and responding to emergency bleeps. Each station was staffed by facilitators experienced in clinical simulation and student training. To introduce added realism, a “bleep system” was integrated into the simulation, simulating the frequent interruptions typical of on-call shifts. This feature aimed to test students’ abilities to manage time and prioritise multiple tasks effectively. Table 1 summarises the content, objectives, and key activities of the nine simulation stations included in the program.

**Table 1:** Summary of Stations: Objectives, Scenarios, and Key Activities. DKA: diabetic ketoacidosis; AKI: acute kidney injury; DNAPCR: Do Not Attempt Cardiopulmonary Resuscitation; ABCDE assessment: Airway, Breathing, Circulation, Disability, Exposure (ABCDE) assessment; MCCD: medical certificate of cause of death

Station	Objectives	Key Activities
Station 1: Managing an acutely unwell patient with anaphylaxis	Analyse acutely unwell patients using a structured ABCDE assessment, implement initial management and appropriate resuscitation and escalate cases effectively using structured communication	Perform initial ABCDE assessment, initiate and reassess management and resuscitate patient appropriately, document and escalate appropriately, Communicate with family and friends
Station 2: Clerking and initial management of a breathless patient	Obtain comprehensive medical history, conduct clinical examinations, generate differential diagnoses, formulate management strategies and complete initial admission documentation	Conduct history and examination, formulate differentials, initiate investigations and treatment, and document findings
Station 3: Fluid and insulin prescribing in DKA	Interpret diabetic ketoacidosis care bundles, prescribe fluids and insulin safely, and document treatment plans according to guidelines.	Prescribe fluids and insulin according to DKA care bundle, document care plan
Station 4: Blood transfusion in a patient with upper gastrointestinal bleed	Label blood samples accurately, prescribe appropriate blood products safely, and obtain informed consent for transfusions.	Draw blood samples, prescribe blood products, document transfusion process, consent and explain process of transfusion
Station 5: Verification of death and documentation	Verify death using clinical and legal standards, record findings comprehensively, and explain the process to bereaved families.	Follow guidelines for verifying death, complete the MCCD, communicate with family
Station 6: Communication for inter-specialty referrals	Summarise patient cases using the Situation, Background, Assessment, Recommendation (SBAR) framework, and record outcomes of discussions and management plans.	Review CT scan report, summarise case, communicate with surgical registrar, and document discussion
Station 7: Safe prescribing in AKI	Evaluate patient conditions Interpret blood tests and recognise AKI. Adjust prescriptions to prevent adverse effects, and document changes clearly.	Interpret blood tests, recognise AKI, adjust prescriptions for nephrotoxic medications, document care plan
Station 8: Hyperkalaemia management – blood results interpretation and management	Identify hyperkalaemia using blood tests and ECG results, initiate emergency management, and document findings and reassessment plans.	Interpret blood and ECG results, initiate hyperkalaemia treatment, and escalate appropriately if needed
Station 9: DNACPR discussion with patient and family	Explain DNACPR decisions to families with compassion, address concerns about end-of-life care, and record discussions in medical notes.	Review notes, update patient and family with condition, explain and discuss DNACPR, address concerns, and document discussion

Pre- and post-session questionnaires

Pre- and post-session anonymous questionnaires were electronically filled. They assessed changes in students' self-reported confidence, prioritisation skills, and perceived preparedness for clinical practice amongst other aspects. Questions measured self-reported confidence scores across variable domains on a Likert scale. Q1-Q22 (1 = not confident at all, 2 = slightly not confident, 3 = neither confident or unconfident, 4 = confident, 5 = very confident). Q23-Q25 (1 = strongly disagree, 2 = disagree, 3 = neither agree or disagree, 4 = agree, 5 = strongly agree). Questions included in the questionnaires are in the table below (Table 2). 

**Table 2:** Pre- and Post-Session Questionnaire. Pre-Session Questionnaire included Q1-Q22. Post-Session Questionnaire included Q23-Q25. SBAR: Situation, Background, Assessment, Recommendation; DNACPR: Do Not Attempt Cardiopulmonary Resuscitation; FY1: Foundation Year 1

Number	Question
1	How confident do you feel about starting your job as a foundation doctor
2	How confident do you feel about working your first on-call shift
3	How confident do you feel in communicating with patients and colleagues
4	How confident do you feel in making clinical decisions under pressure
5	How confident are you in your ability to prioritise tasks during on-call shifts
6	How confident are you in your abilities to handle accurate documentation and record-keeping
7	How confident are you in your ability to perform core clinical skills during on-call situations
8	How confident do you feel in your ability to interpret laboratory and imaging results during on-call shifts
9	How confident do you feel in knowing how to seek help when needed (I.e correct escalation pathways)
10	How confident do you feel in obtaining a patient's medical history and conducting a clinical examination
11	How confident do you feel in managing an acutely unwell patient
12	How confident are you in your ability to prescribe fluids
13	How confident do you feel in managing a patient with an acute bleed
14	How confident are you in verifying and documenting a patient's death
15	How confident do you feel in communicating effectively using SBAR
16	How confident are you in your ability to prescribe medications safely and correctly
17	How confident are you in having a DNACPR discussion with patients and family members
18	How confident do you feel in acting upon abnormal results
19	How confident do you feel in starting initial management for a patient
20	How confident do you feel in escalating to seniors using SBAR
21	How confident are you in your ability to be part of the cardiac arrest team
22	How confident do you feel in starting initial management for an unwell patient
23	This event has made me more familiar with the typical on-call responsibilities and challenges (post-session only)
24	This event has helped me develop skills needed for on-call duties (post-session only)
25	This event has made me more prepared for on-calls as a FY1 doctor (post-session only)

Quantitative analysis of questionnaire data

Quantitative data from the Likert-scale questionnaires were analysed using descriptive and inferential statistics to evaluate changes in students' confidence, prioritisation abilities, and preparedness for on-call duties. For each question, the mean pre-session and post-session scores, along with their standard deviations and 95% confidence intervals, were calculated to summarise the data.

Statistical analysis

Data from the pre-session and post-session questionnaires were analysed to assess changes in students' self-reported confidence and preparedness across various competencies. Since individual pre-session and post-session responses were collected anonymously and could not be paired, and the data were not normally distributed (Shapiro-Wilk test, p-value < 0.001), non-parametric statistical methods were employed.

The Mann-Whitney U test was selected to compare the distribution of scores between the pre-session and post-session groups for each questionnaire item. This test is appropriate for comparing two independent samples when the data are ordinal and not normally distributed. All tests were conducted as two-tailed to detect significant differences in either direction.

To evaluate the practical significance of the findings, effect sizes were calculated using the rank biserial correlation coefficient (rₛ). It measures and quantifies the strength and direction of the association between two variables. rₛ values range from -1 to +1, where values closer to +1 or -1 indicate stronger associations and values near 0 suggest little to no association. Given that our questionnaire responses are ordinal and do not assume a normal distribution, rₛ offers an appropriate and meaningful measure of effect size, allowing us to effectively interpret the strength of the observed changes in confidence scores. The magnitude of rₛ was interpreted as follows: 0.1 to 0.3 indicates a small effect, 0.3 to 0.5 a moderate effect, and 0.5 and above a large effect.

## Results

A total of 70 students completed the pre-session questionnaire, and 72 completed the post-session questionnaire, assessing their self-reported confidence, prioritisation skills, and preparedness for clinical practice across various competencies. Statistical analysis revealed significant improvements across all areas following the SOC program.

**Figure 1:**
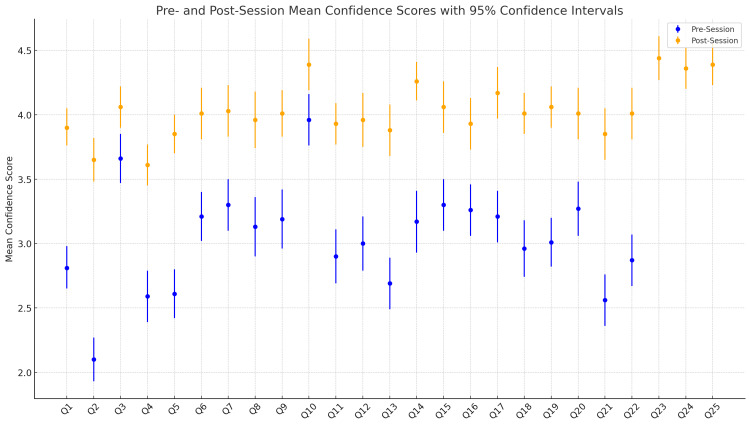
Pre- And Post-Session Mean Confidence Scores With 95% Confidence Intervals

Figure 1 illustrates the mean confidence scores with 95% confidence intervals for all 25 questions, comparing pre- and post-session responses. For Questions 1-22, both pre- and post-session means are displayed, while Questions 23-25, exclusive to the post-session, represent the perceived impact of the simulation program on overall preparedness. Notable improvements are observed across nearly all questions, with the post-session confidence intervals generally shifted upward, indicating increased confidence following the intervention. This visual highlights the program’s impact on self-perceived confidence levels (Figure 1). 

Table 3 provides a detailed breakdown of the statistical analysis for each question, listing pre- and post-session mean scores with 95% confidence intervals (CI), p-values (p), and rank biserial correlation coefficients (rₛ) to measure effect size (Table 3). 

**Table 3:** Comparison of Pre- and Post-Session Mean Confidence Scores, p-Values, and Rank Biserial Correlation Coefficients (rₛ) p-values <0.05 are considered significant. r_s_ (0.1 to 0.3: Small effect size, 0.3 to 0.5: Moderate effect size, 0.5 and above: Large effect size). Pre-mean and post-mean values are based on Likert-scale measurements as below. Q1-Q22 (1 = not confident at all, 2 = slightly not confident, 3 = neither confident or unconfident, 4 = confident, 5 = very confident). Q23-Q25 (1 = strongly disagree, 2 = disagree, 3 = neither agree or disagree, 4 = agree, 5 = strongly agree).

Question	Pre-mean	Post-mean	p	rₛ
Q1 - How confident do you feel about starting your job as a foundation doctor	2.81 (95% CI: 2.65 - 2.68)	3.90 (95% CI: 3.76 - 4.05)	<0.001	0.71
Q2 - How confident do you feel about working your first On-Call shift	2.10 (95% CI: 1.93 - 2.27)	3.65 (95% CI: 3.48 - 3.82)	<0.001	0.83
Q3 - How confident do you feel in communicating with patients and colleagues	3.66 (95% CI: 3.47 - 3.85)	4.06 (95% CI: 3.90 - 4.22)	0.002	0.28
Q4 - How confident do you feel in making clinical decisions under pressure	2.59 (95% CI: 2.39 - 2.79)	3.61 (95% CI: 3.45 - 3.77)	<0.001	0.62
Q5 - How confident are you in your ability to prioritise tasks during on-call shifts	2.61 (95% CI: 2.42 - 2.80)	3.85 (95% CI: 3.70 - 4.00)	<0.001	0.74
Q6 - How confident are you in your abilities to handle accurate documentation and record-keeping	3.21 (95% CI: 2.99 - 3.43)	4.01 (95% CI: 3.85 - 4.17)	<0.001	0.47
Q7 - How confident are you in your ability to perform core clinical skills during on-call situations	3.3 (95% CI: 3.08 - 3.52)	4.03 (95% CI: 3.87 - 4.19)	<0.001	0.45
Q8 - How confident do you feel in your ability to interpret laboratory and imaging results during on-call shifts	3.13 (95% CI: 2.92 - 3.34)	3.96 (95% CI: 3.79 - 4.13)	<0.001	0.51
Q9 - How confident do you feel in knowing how to seek help when needed (I.e Correct escalation pathways)	3.19 (95% CI: 2.96 - 3.42)	4.01 (95% CI: 3.83 - 4.19)	<0.001	0.49
Q10 - How confident do you feel in obtaining a patient's medical history and conducting a clinical examination	3.96 (95% CI: 3.75 - 4.17)	4.39 (95% CI: 4.25 - 4.53)	0.003	0.27
Q11 - How confident do you feel in managing an acutely unwell patient	2.90 (95% CI: 2.69 - 3.11)	3.93 (95% CI: 3.77 - 4.09)	<0.001	0.60
Q12 - How confident are you in your ability to prescribe fluids	3.00 (95% CI: 2.75 - 3.25)	3.96 (95% CI: 3.79 - 4.13)	<0.001	0.51
Q13 - How confident do you feel in managing a patient with an acute bleed	2.69 (95% CI: 2.47 - 2.91)	3.88 (95% CI: 3.71 - 4.05)	<0.001	0.66
Q14 - How confident are you in verifying and documenting a patient's death	3.17 (95% CI: 2.93 - 3.41)	4.26 (95% CI: 4.11 - 4.41)	<0.001	0.61
Q15 - How confident do you feel in communicating effectively using Situation, Background, Assessment, Recommendation (SBAR)	3.30 (95% CI: 3.10 - 3.50)	4.06 (95% CI: 3.91 - 4.21)	<0.001	0.50
Q16 - How confident are you in your ability to prescribe medications safely and correctly	3.26 (95% CI: 3.04 - 3.48)	3.93 (95% CI: 3.76 - 4.10)	<0.001	0.40
Q17 - How confident are you in having a Do Not Attempt Cardiopulmonary Resuscitation (DNACPR) discussion with patient and family members	3.21 (95% CI: 2.97 - 3.45)	4.17 (95% CI: 4.00 - 4.34)	<0.001	0.54
Q18 - How confident do you feel in acting upon abnormal results	2.96 (95% CI: 2.77 - 3.15)	4.01 (95% CI: 3.85 - 4.17)	<0.001	0.67
Q19 - How confident do you feel in starting initial management for a patient	3.01 (95% CI: 2.82 - 3.20)	4.06 (95% CI: 3.90 - 4.22)	<0.001	0.63
Q20 - How confident do you feel in escalating to seniors using Situation, Background, Assessment, Recommendation (SBAR)	3.27 (95% CI: 3.05 - 3.49)	4.01 (95% CI: 3.82 - 4.20)	<0.001	0.45
Q21 - How confident are you in your ability to be part of the cardiac arrest team	2.56 (95% CI: 2.31 - 2.81)	3.85 (95% CI: 3.65 - 4.05)	<0.001	0.62
Q22 - How confident do you feel in starting initial management for an unwell patient	2.87 (95% CI: 2.65 - 3.09)	4.01 (95% CI: 3.85 - 4.17)	<0.001	0.65
Q23 - This event has made me more familiar with the typical on-call responsibilities and challenges (post-session only)	N/A	4.44 (95% CI: 4.27 - 4.61)	N/A	N/A
Q24 - This event has helped me develop skills needed for on-call duties (post-session only)	N/A	4.36 (95% CI: 4.20 - 4.52)	N/A	N/A
Q25 - This event has made me more prepared for on-calls as a Foundation Year 1 (FY1) doctor (Post-session only)	N/A	4.39 (95% CI: 4.23 - 4.55)	N/A	N/A

Overall confidence and prioritisation skills

Significant gains were observed in questions related to students’ overall confidence and ability to prioritise tasks. 

**Figure 2:**
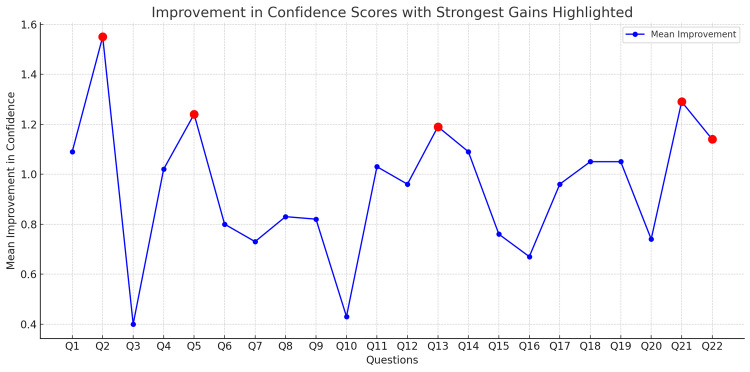
Improvement In Confidence Scores With Strongest Gains Highlighted Q2 - How confident do you feel about working your first on-call shift Q5 - How confident are you in your ability to prioritise tasks during on-call shifts Q13 - How confident do you feel in managing a patient with an acute bleed Q21 - How confident are you in your ability to be part of the cardiac arrest team Q22 - How confident do you feel in starting initial management for an unwell patient

Q1, assessing confidence in starting as a foundation doctor, mean scores increased from 2.81 (95% CI: 2.65-2.98) pre-session to 3.90 (95% CI: 3.76-4.05) post-session, an improvement of 1.1 points (p < 0.001) with a large effect size (rₛ = 0.71), indicating a robust enhancement in perceived readiness. The largest single improvement was seen in Q2, which evaluated confidence in handling the first on-call shift. Scores rose from 2.10 (95% CI: 1.93-2.27) to 3.65 (95% CI: 3.48-3.82), an improvement of 1.55 points (p < 0.001) (Figure 2). The effect size (rₛ = 0.83) indicates a substantial shift in students’ confidence in this critical early-career skill. Additionally, confidence in prioritising tasks during on-call shifts, measured by Q5, increased from a pre-session mean of 2.61 (95% CI: 2.42-2.80) to a post-session mean of 3.85 (95% CI: 3.70-4.00), reflecting an improvement of 1.24 points (p < 0.001) (Figure 2). This change similarly had a large effect size (r_s_=0.74), underscoring the program’s impact on prioritisation skills. Confidence in making clinical decisions under pressure, as evaluated by Q4, also increased from 2.59 (95% CI: 2.39-2.79) pre-session to 3.61 (95% CI: 3.45-3.77) post-session (p<0.001), with an effect size of r_s_=0.62 (Figure 1).

Clinical competencies and communication

In competencies related to clinical skills and communication, significant increases in confidence were also reported.

Q14, which assessed confidence in verifying and documenting a patient’s death, mean scores rose from 3.17 (95% CI: 2.93-3.41) pre-session to 4.26 (95% CI: 4.11-4.41) post-session (p<0.001, r_s_=0.61). Confidence in interpreting laboratory and imaging results, assessed by Q8, improved from 3.13 (95% CI: 2.90-3.36) to 3.96 (95% CI: 3.74-4.18) post-session (p<0.001, r_s_=0.51). Confidence in managing acutely unwell patients, measured by Q11, increased from 2.90 (95% CI: 2.69-3.11) pre-session to 3.93 (95% CI: 3.77-4.09) post-session (p<0.001, r_s_=0.55). While confidence in communicating with patients and colleagues (Q3) also improved, the increase was more modest, from 3.66 (95% CI: 3.47-3.85) pre-session to 4.06 (95% CI: 3.90-4.22) post-session (p=0.002), with a smaller effect size (r_s_=0.28). Q9, which assessed students' confidence in escalating issues and knowing how to seek help, showed a significant improvement from 3.19 (95% CI: 2.96 - 3.42) pre-session to 4.01 (95% CI: 3.83 - 4.19)post session (p<0.001, r_s_=0.49) (Figure 1).

Overall preparedness and final reflections

In terms of overall preparedness, initial management and escalation, students also reported substantial increases in confidence. For example, Q19, which assessed confidence in starting initial management for a patient, rose from 3.01 (95% CI: 2.82-3.20) pre-session to 4.06 (95% CI: 3.90-4.22) post-session (P<0.001, r_s_=0.63), demonstrating a notable increase in perceived preparedness for managing early clinical tasks. Similarly, confidence in acting upon abnormal results, as assessed by Q18, improved from 2.96 (95% CI: 2.77-3.15) to 4.01 (95% CI: 3.85-4.17) post-session (p<0.001, r_s_=0.67). Additionally, confidence in knowing when to seek help, including understanding escalation pathways (Q9), increased from 3.19 (95% CI: 2.96-3.42) pre-session to 4.01 (95% CI: 3.83-4.19) post-session (p<0.001, r_s_=0.49) (Figure 1).

In the post-session exclusive questions, students indicated substantial perceived gains in preparedness for on-call duties. Q23, which queried overall familiarity with on-call responsibilities, yielded a high mean score of 4.44 (95% CI: 4.27-4.61). Similarly, Q24 and Q25, which evaluated skill development for on-call duties and overall readiness to begin working as a doctor, scored means of 4.36 (95% CI: 4.20-4.52) and 4.39 (95% CI: 4.23-4.55), respectively. These high post-session scores reflect a strong sense of preparedness and improved confidence among students (Figure 1).

Effect sizes 

**Figure 3:**
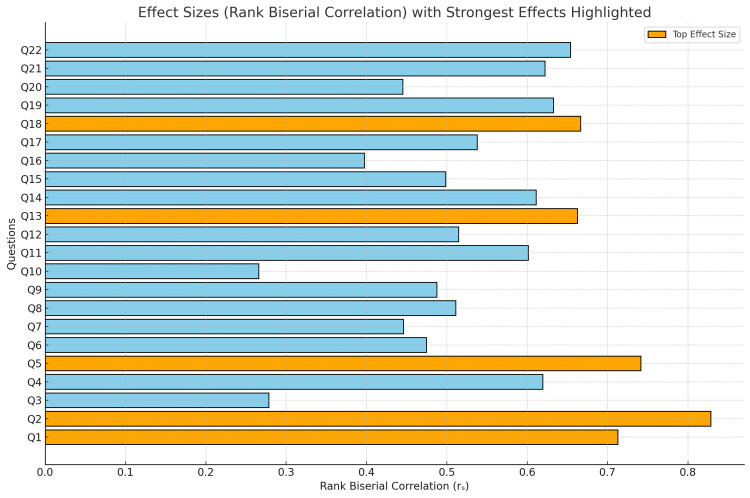
Effect Sizes (Rank Biserial Correlation) With Strongest Effects Highlighted Q1 - How confident do you feel about starting your job as a foundation doctor Q2 - How confident do you feel about working your first On-Call shift Q5 - How confident are you in your ability to prioritise tasks during on-call shifts Q13 - How confident do you feel in managing a patient with an acute bleed Q18 - How confident do you feel in acting upon abnormal results

The rank biserial correlation coefficients (rₛ) indicated moderate to large effect sizes across competencies, with values ranging from 0.44 to 0.83 in key areas. Figure 3 demonstrates a summary of effect sizes with the strongest highlighted. Notable effect sizes were observed for questions assessing confidence in prioritisation skills, handling on-call scenarios, and in starting work as a foundation doctor. (e.g., Q2 with r_s_=0.83, Q5 with r_s_=0.74). This indicates a substantial impact and practical effect on participants' self-reported confidence (Figure 3).

The lowest increases in mean scores were observed in Q3 (confidence in communicating with patients and colleagues) and Q10 (confidence in obtaining a patient's medical history and conducting a clinical examination). Q3 improved by only 0.40 points, from a pre-session mean of 3.66 (95% CI: 3.47 - 3.85) to 4.06 (95% CI: 3.90 - 4.22) post-session (p=0.002, rs=0.28) while Q10 increased by 0.43 points (p=0.003, r_s_=0.027)(Figures 1, 2). In summary, there were statistically significant improvements in self-reported confidence in all areas assessed. The highest gains were observed in competencies essential for on-call duties, such as task prioritisation, initial patient management, and escalation, reinforcing the program’s value as an educational intervention that prepares final-year students for the clinical demands of foundation practice.

## Discussion

The transition from medical student to practising doctor is widely recognised as a challenging period, marked by the need to rapidly adapt to new responsibilities, particularly during on-call duties, which require rapid decision-making and efficient task prioritisation [10-12,19]. Our study aimed to evaluate the impact of a one-day simulation-based "on-call" training program on final-year medical students' confidence, prioritisation skills, and preparedness for clinical practice. The findings demonstrate significant improvements across multiple competencies, highlighting the effectiveness of simulation in bridging the gap between theoretical knowledge and practical application.

One of the most critical skills for new doctors is the ability to prioritise tasks effectively, especially during on-call shifts, where swift decision-making is essential [18,19,22]. The significant increase in confidence reported in Q5 (task prioritisation), with a pre- to post-session mean change from 2.61 to 3.85 (p < 0.001, rₛ = 0.74), aligns with previous findings emphasising the importance of structured training in enhancing time management and prioritisation abilities [18]. Structured training in prioritisation can improve performance under time constraints, helping to prepare students for real-world clinical responsibilities [18]. Prioritisation has also been identified as a high-impact competency that is well-suited to simulation-based education, highlighting its role in enhancing decision-making under pressure [19]. Our results support these findings, suggesting that simulation-based programs can effectively enhance prioritisation skills, which are often underemphasised in traditional curricula [18,19,22].

The largest improvement was observed in Q2, assessing confidence in working the first on-call shift, which increased from 2.10 to 3.65 (p < 0.001, rₛ = 0.83). This substantial gain reflects the effectiveness of the simulation in addressing anxieties related to on-call responsibilities. Similar findings were reported by Carpenter et al., who found that simulation training made new graduates feel more prepared for the realities of professional practice [19].

The marked improvements in confidence related to managing acutely unwell patients (Q11) and knowing when to seek help (Q9) address areas where new graduates often feel underprepared [11,12]. Q11 saw an increase from 2.90 to 3.93 (p < 0.001, rₛ = 0.60), while Q9 rose from 3.19 to 4.01 (p < 0.001, rₛ = 0.49). Sturman et al. highlighted that junior doctors experience a "steep learning curve" in acute patient management and escalation of care [11]. By simulating high-pressure clinical scenarios, our program provided a safe environment for students to practice critical decision-making and communication skills, which are essential for patient safety [1,4]. This experiential learning approach aligns with the findings of Banerjee et al., who demonstrated that simulation-based curricula enhance teamwork and communication skills among medical students [4]. The results highlight the potential of simulations to address these high-stress areas by providing realistic practice that builds confidence in critical decision-making and collaboration skills.

The integration of a "bleep system" in our simulation added realism by mimicking frequent interruptions typical of on-call shifts. This aligns with the recommendations by Gaba, who emphasised the importance of incorporating realistic elements into simulation to enhance learning outcomes [23]. By exposing students to the multitasking demands of on-call duties, the simulation provided a safe environment for them to test their abilities to manage interruptions and maintain task focus, skills critical for patient safety and effective clinical practice.

Interestingly, the smallest increases in confidence were observed in areas where students already felt relatively confident pre-session, such as communication with patients and colleagues (Q3), obtaining a patient's medical history and conducting a clinical examination (Q10). Q3 improved by 0.40 points (p = 0.002, rₛ = 0.28), and Q10 increased by 0.43 points (p = 0.003, rₛ = 0.27). This suggests that simulation may have the greatest impact on competencies where students initially feel less prepared [10]. Teagle et al. noted that targeted preparatory programs can significantly boost confidence in specific areas, facilitating a smoother transition into clinical roles [10]. Our findings reinforce the importance of tailoring simulation experiences to address identified gaps in students' preparedness.

An important consideration is the potential impact of simulation training on patient outcomes. While our study focused on self-reported confidence and preparedness, previous research has demonstrated that simulation-based education can lead to tangible improvements in patient care [9]. Barsuk et al. showed that simulation-based mastery learning significantly reduced catheter-related bloodstream infections in a clinical setting [9]. This suggests that the benefits of simulation extend beyond learner preparedness to actual enhancements in patient safety and quality of care. Therefore, incorporating simulation into medical education not only equips students with the necessary skills but may also contribute to better patient outcomes in their future practice.

Despite the clear benefits, it is important to acknowledge the limitations of simulation-based training. While simulation provides a controlled environment for skill development, it cannot entirely replicate the unpredictability and complexity of actual clinical practice [11,25]. Kneebone et al. argued that while simulation is valuable, it should complement, not replace, real clinical experiences [25]. The controlled nature of simulation may not fully prepare students for the nuanced, dynamic challenges encountered in live clinical settings, particularly under high pressure or with limited resources [19,26].

Overall, students reported feeling significantly more confident and more prepared to begin their clinical practice. This highlights the potential use of structured simulation in combination with practical experiences to connect foundational knowledge with hands-on clinical application. This is consistent with the literature advocating for the integration of simulation into medical education to improve readiness for clinical practice [19,22,23]. Ingrassia et al. emphasised that simulation allows for the prioritisation of critical competencies, ensuring that students are better equipped to handle real-world challenges [22]. Simulation-based training can significantly enhance students’ preparedness by allowing them to practice essential skills in a safe and controlled environment [10,19,22]. Our results align with this, showing that participation in a one-day on-call simulation led to substantial improvements in students' self-reported confidence and readiness across multiple competencies.

Targeted preparation programs can significantly boost confidence and skill acquisition, particularly in areas like task prioritisation and managing acutely ill patients. Such programs not only prepare students technically but also bolster their psychological readiness for the demands of early clinical practice, facilitating a smoother transition into foundation roles [13]. Although simulation cannot fully replicate the unpredictability and accountability of actual clinical practice, it can act as an effective supplement by allowing repeated practice and reflection in key areas that reduce initial on-call stress and improve task prioritisation and escalation skills [22].

Limitations

There are several limitations that should be considered when interpreting the results. First, the sample size, although reflective of the student cohort, may limit the generalisability of findings to broader populations or different educational settings. Additionally, our study relied on self-reported confidence measures, which, although useful, may not directly correlate with actual clinical performance [13]. Boakes and Shah highlighted the need for objective assessments to validate the impact of preparatory courses on clinical competence [13].

Another limitation is that the post-session questionnaire was administered immediately after the simulation, measuring only the immediate impact on confidence without assessing long-term retention of skills or knowledge. Future iterations could incorporate follow-up assessments to evaluate whether these confidence gains persist over time and how well they translate into clinical competency during actual on-call duties.

Since individual pre-session and post-session responses were collected anonymously and could not be paired, the data were treated as independent samples. This prevented us from performing paired statistical analyses, which may have provided more precise estimates of the program's impact on individual students.

These limitations suggest the need for additional research, particularly longitudinal studies that examine the retention of skills and confidence over time and explore how well simulation training prepares students for on-call responsibilities in unpredictable clinical environments.

## Conclusions

The one-day simulation-based "On-Call" training program significantly enhanced final-year medical students' confidence, prioritisation skills, and preparedness for clinical practice. The findings support the integration of targeted simulation experiences into medical curricula to address specific areas where students feel underprepared. By providing realistic and high-pressure clinical scenarios, simulation allows students to practice and refine both technical and non-technical skills in a safe environment. While simulation is a powerful educational tool, it should be used in conjunction with real clinical experiences to fully prepare students for the complexities of medical practice. Further research is necessary to evaluate the long-term impact of simulation training on clinical performance and patient outcomes.
